# Heart Alterations after Domoic Acid Administration in Rats

**DOI:** 10.3390/toxins8030068

**Published:** 2016-03-10

**Authors:** Andres C. Vieira, José Manuel Cifuentes, Roberto Bermúdez, Sara F. Ferreiro, Albina Román Castro, Luis M. Botana

**Affiliations:** 1 Departamento de Farmacología, Facultad de Veterinaria, Universidad de Santiago de Compostela, Lugo 27002, Spain; andres.crespo.vieira@gmail.com (A.C.V.); sara.fernandez.ferreiro@usc.es (S.F.F.); 2 Departamento de Anatomía, Facultad de Veterinaria, Universidad de Santiago de Compostela, Lugo 27002, Spain; roberto.bermudez@usc.es; 3 Rede de Infraestruturas de Apoio á Investigación e ao Desenvolvemento Tecnolóxico (RIADT) Lugo, Universidad de Santiago de Compostela, Lugo 27002, Spain; a.roman@usc.es

**Keywords:** cardiotoxicity, domoic acid, electron microscopy, immunohistochemistry, phycotoxin

## Abstract

Domoic acid (DA) is one of the best known marine toxins, causative of important neurotoxic alterations. DA effects are documented both in wildlife and experimental assays, showing that this toxin causes severe injuries principally in the hippocampal area. In the present study we have addressed the long-term toxicological effects (30 days) of DA intraperitoneal administration in rats. Different histological techniques were employed in order to study DA toxicity in heart, an organ which has not been thoroughly studied after DA intoxication to date. The presence of DA was detected by immunohistochemical assays, and cellular alterations were observed both by optical and transmission electron microscopy. Although histological staining methods did not provide any observable tissue damage, transmission electron microscopy showed several injuries: a moderate lysis of myofibrils and loss of mitochondrial conformation. This is the first time the association between heart damage and the presence of the toxin has been observed.

## 1. Introduction

Domoic acid (DA) is produced by diatoms, microscopic algae characterized by the presence of a cell wall made of hydrated silicon dioxide named frustule [1,2]. Diatoms species belong to a group of microorganisms able to produce marine toxins (phycotoxins), responsible for the appearance of harmful algal blooms [3,4].

When DA is present at high concentrations in diatoms, this toxin can be bio-accumulated in shellfish and finfish, causing the poisoning of seabirds, marine mammals or humans [5]. In the year 1987, DA was responsible for four deaths and the illness of more than 100 people after consuming blue mussels (*Mytilus edulis*) harvested in the Cardigan Bay of Prince Edward Island, Canada [6,7,8]. The symptomatology comprised three kinds of signs: gastrointestinal (nausea, vomiting, abdominal cramps and diarrhoea), cardiovascular (unstable blood pressure and arrhythmias), and neurological signs (disorientation, confusion, headaches, hallucinations, coma, seizures and memory impairment) [6,7,8]. Memory impairment led to the denomination of this condition as amnesic shellfish poisoning (ASP) [6,7,8] .

DA is a hydrophilic acid whose structure is very similar to kainic acid (KA), being both DA and KA analogues of glutamate (Glu). Glu is the main excitatory neurotransmitter in the brain, playing a key role in long-term potentiation [9,10,11]. DA has high affinity for the glutamate receptors (GluRs) subtypes alpha-amino-3-hydroxy-5-methyl-4-isoxazolepropionic acid (AMPA) and KA receptors [12,13]. The binding of DA to receptors provokes an increase of calcium (Ca^2+^) levels, causing the release of Glu to the extracellular space, and the activation of *N*-methyl-d-aspartate (NMDA) receptors [14,15]. The combined action of Glu release and the increase of intracellular (Ca^2+^) levels produces Reactive Oxide Species (ROS) and the activation of phospholipases, protein kinase C, proteases, protein-phosphatases, caspases and nitric oxide synthases [14,15,16,17]. The histological consequences of these cellular alterations comprise astrocytosis, cytoskeletal disarrangement and, finally, cell death.

Numerous *in vivo* assays, developed principally in mice and rats, have described DA neuropathological effects. The brain was the main affected tissue, and the lesions were mainly located in the hippocampal area [17,18,19,20], according to the high concentration of KA receptors in this area [13,21,22,23]. Other Central Nervous System (CNS) areas were also affected by this neurotoxin, such as amygdala, olfactory bulb and hypothalamus [24,25] .

Although DA is mainly known due to its neurotoxic effects, besides lesions described in the CNS, heart affectation was also reported after some DA intoxications. In wildlife the most severe damage was found in intoxicated California sea lions (*Zalophus californianus*), consistent with necrosis and oedemas in the base of the heart, with mild lesions toward the apex [26,27]. Important lesions have been also described in southern sea otters (*Enhydra lutris nereis*), comprising of multifocal myocardial necrosis associated with myocardial haemorrhage [28]. The cardiac alterations caused by DA may be a possible explanation for the cardiac effects observed in 1987 [6] .

Experimental assays with rats also showed important cardiac damage, that was composed of cell vacuolization, fibrosis, inflammatory infiltrates (macrophages and leukocytes), and necrosis in the subendocardium, papillary muscle, and septal regions around the left ventricle [29]. Due to the presence of GluRs in the heart (concretely they are expressed within the conducting system, in the cardiac intramural nerve fibers and ganglia cells [30,31]), it seems that DA exerts its cardiotoxic effects through a direct way. Nevertheless, the *in vitro* assays did not show any damage in the cardiomyocytes exposed to the toxin [32]. Hence, DA might exert its influence on heart tissue by an indirect way [29] .

Therefore, the mechanism of action through which the DA exerts its cardiotoxicity remains unknown. The objective of the present study was to assess the potential cardiotoxic effects of DA, and to elucidate if this toxin alters cardiac tissue through a direct binding of DA to cardiac GluRs or by affecting neurons of the limbic system. For this purpose, long-term histological alterations in heart (30 days) were evaluated, after the intraperitoneal (i.p.) administration of a single dose of DA in rats. One hour before sacrifice, a new DA dose was administered, in order to detect the toxin by immunohistochemical analysis.

## 2. Results

High-performance liquid chromatography-Ultraviolet/Visible (HPLC-UV) analysis revealed no significant differences between the expected and the actual quantity of DA provided by the supplier laboratory, guaranteeing that the dose administered was 2.5 mg/kg in all the cases .

After first administration of DA, all rats displayed hypoactivity, head shaking, convulsions and scratching. During the first 24 h after toxin administration, most of the rats displayed anorexia (*n* = 8) and/or adipsia (*n* = 7). Four of the nine rats died in a period between 5 and 90 min, showing severe convulsions before death .

After 24 h, adipsia and anorexia were present in many intoxicated rats, one specimen still displayed a slight scratching and two rats showed hematoporphyrin deposits in eyes and nostrils.

Finally, 72 h after DA administration, only two rats showed anorexia, and one of these specimens also displayed adipsia. Hematoporphyrin deposits were not visible in any of the rats. Control animals, injected with physiologic saline solution, did not develop any abnormal symptom. A brief summary of the symptomatology is presented in Table 1.

After the second administration of the toxin, scratching, hypoactivity and convulsions were also noted before sacrifice, being as severe as those observed during the first administration of DA. Due to the limited time before sacrifice, water or food intake were not taken into account .

**Figure 1:**
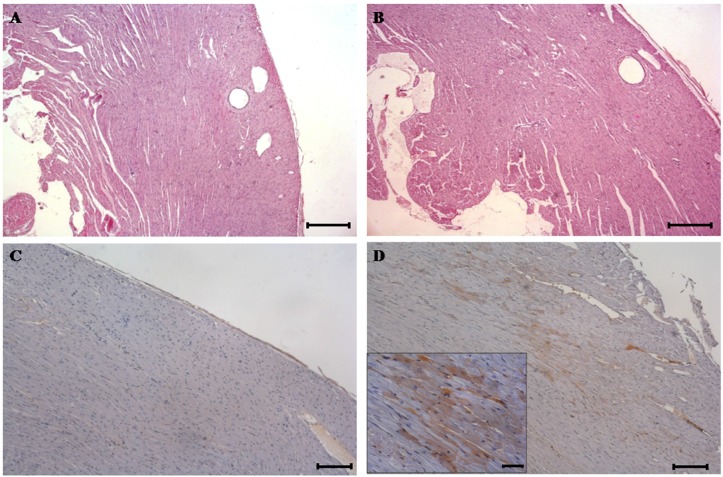
Haematoxylin and eosin (H & E) stained sections of heart tissue from control and domoic acid (DA)-treated rats. (**A**) H & E-stained sections from control rats. (original magnification, ×40, scale bar = 200 μm); (**B**) H & E-stained sections of heart tissue from DA-treated rats showing no visible damage (original magnification, ×40, scale bar = 200 μm); (**C**) DA Immunohistochemistry (IHC) of the heart from control rats. The phycotoxin is absent throughout the tissue. IHC with DA antibody 1:5000 (original magnification, ×100, scale bar = 60 μm); (**D**) DA IHC of heart from DA-treated rats showing immunoreactivity in myocardium. IHC with DA antibody 1:5000 (original magnification,×100, scale bar = 60 μm). The boxed area shows the positive immunostaining located in the cytoplasm of the cardiomyocytes at higher magnifications (original magnification, ×400, scale bar = 20 μm).

Light microscopy of the heart from control (Figure 1A), and treated rats (Figure 1B), including dead animals after the first DA administration, revealed no histological changes.

Immunohistochemistry (IHC) against DA did not show traces of the toxin in control animals (Figure 1C). However, immunoreactivity was observed in DA-treated rats, within cytoplasm of several randomly distributed groups of cardiomyocytes (Figure 1D).

**Figure 2:**
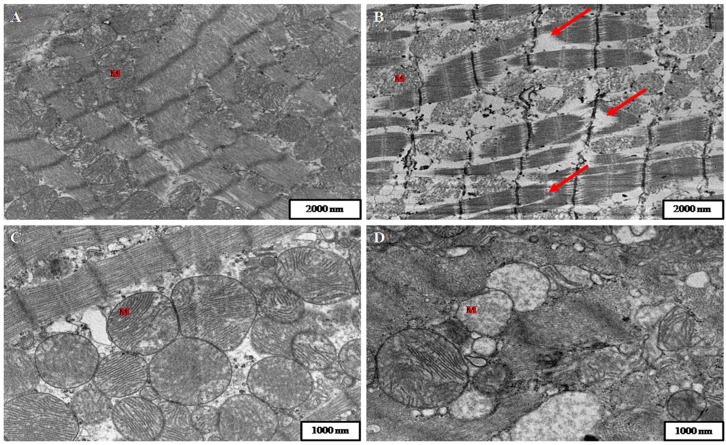
Transmission Electron Microscopy (TEM) of heart samples from control and DA-treated rats. (**A**) Cardiomyocyte from of a control specimen showing the typical arrangement of myofibrils and mitochondria (M); (**B**) Ultrastructure of rat cardiac muscle from a DA-treated rat. A moderate lysis of myofibrils (arrows) and degeneration of mitochondria (M) can be observed; (**C**) Cardiomyocyte from a control specimen showing mitochondria (M) with their usual shape and electron density; (**D**) Cardiomyocyte from DA treated rat showing degenerated mitochondria (M). These organelles had lost their habitual rounded shape, they were less electron dense than mitochondria of control animals and displayed cristolysis.

Employing transmission electron microscopy (TEM), heart from control animals showed a typical myofibrillar arrangement (Figure 2A), while cardiomyocyte degeneration was observed in DA-treated rats, with an evident lack of integrity in the myofibrils which were in disarray (Figure 2B). All myofibril constituents were affected, and only the Z bands remained undamaged. Additionally, mitochondria were less electron dense than the same organelles from control specimens, showing mitochondrial swelling and cristolysis, that is, the lysis and breakage of mitochondrial cristae. Mitochondrial disposition was also altered; while control specimens showed an organized arrangement and mitochondria were clearly visible, with a normal round shape (Figure 2C), DA-treated rats displayed mitochondria in large accumulations through the whole cell and several were degenerated (Figure 2D).

## 3. Discussion

Rodents, particularly mice, are the species most employed for assessing toxicity in *in vivo* assays, far more than other organisms such as fishes, cats or monkeys. Rats are still used in some toxicological tests, such as the DA toxicological studies. The reasons are that rats are more sensible to DA than mice, and the lethality observed in rats is closer to that observed in human beings [33].

Previous studies showed a similar symptomatology in both rodent species: scratching, hypoactivity, convulsions and aggressiveness. [18,34,35,36,37,38,39,40,41,42,43]. When different memory tests were employed, a short-term memory loss was frequently observed [20,44,45]. Behavioral observations obtained in the present study concur with the typical symptomatology described in the bibliography. Additionally, the adipsia, although not as frequent as the other symptoms, has been also observed in our previous works [46].

There is some controversy about the cardiac effects elicited after DA administration. Cardiac damage (concretely, myocardial fibrosis, nuclear hyperplasia and hypertrophy), was described both in sea otters and sea lions [26,27,28]; although due to the fact that these animals were naturally affected, there is a paucity of data about the DA dose able to cause these alterations, or how long the affected animals were exposed to the toxin. Vranyac-Tramoundanas *et al.* reported cardiac damage in rats after DA intrahippocampal and i.p. administration, mainly consistent of cardiomyocite vacuolization, myofiber loss and inflammatory cell infiltrate [29]. Nevertheless, previously they did not show any observable damage in isolated cardiomyocytes exposed to the toxin [32] .

Previous studies accomplished in our laboratory involved studying the presence of the toxin or cardiac damage in rats after short time lapses of exposition [47]. Specimens were sacrificed between 6 and 24 h after toxin i.p. administration [47]. Even so, DA was not detected and no damage was observed [47]. Therefore, it is possible that DA damage can only be seen some time after toxin administration (at least more than 24 h).

In the present study, heart samples did not show any histological alteration on light microscopy. However, immunohistochemical analysis detected DA presence into the cytoplasm of the cardomyocytes. Hence, the DA was able to reach cardiac tissue after i.p. exposition and it was able to traverse the cell membrane. This finding suggests that DA could cause disturbances in a direct way and not just indirectly through the hippocampus and hypothalamic paraventricular nucleus, which project into the cardiac regulatory centers [32]. The existence of GluRs in heart may explain the DA affinity for this tissue [30,31]. Nevertheless, despite DA detection in cardiomyocytes, it is important correlating this fact with the damage produced by the toxin. Even though conventional staining methods did not show observable injuries in heart tissue, with TEM analysis several injuries in cardiac cells were detected .

The injuries in mitochondria may have important implications due to their role in energetic metabolism. Previous *in vitro* assays performed with cardiac mitochondria isolated from DA-treated rats revealed alterations in the mitochondrial electron transport complex [32]. A reduction in activity of complexes I and II–III was noted in those studies after DA incubation. It is known that toxins that interfere with oxidative phosphorylation or electron transport in mitochondrial cristae will rapidly lead to ATP depletion and swelling of these organelles, which might affect the proper heart functioning [48].

The mitochondrial dysfunctions are implied in the pathology of a wide variety of common diseases, including cardiac failure, considering that important decreases in the activity of these enzymatic complexes were ascertained in cardiac failure [49,50,51].

Although heart tissue damage caused by DA was mild, the moderate myofibril lysis may affect the normal functioning of cardiomyocytes, contributing to the cardiac damage .

The moderate myofibril lysis observed may have important implications: DA alteration may affect the normal functioning of cardiomyocytes and may contribute in some way to the cardiac arrest. Although heart tissue damage was mild, it may affect, to a certain degree, the normal functioning of the cardiomyocytes.

Mitochondrial injury and myofibril lysis might be closely related. Bayeva and Ardehali showed that mitochondria injuries could cause an important ROS generation which, in turn, may cause a preferential oxidation of myofibrillar proteins and the disruption of actin-myosin interactions [52]. The subsequent sarcomere alterations may promote the impairment of heart normal function.

This is the first time that correlation between the presence of DA and the observation of cardiac damage in rats is described. Although the heart damage observed was mild, this damage proves that DA is able to cause some kind of cardiotoxicity after its administration in rats. Despite the detection of the toxin in cardiac tissue by immunohistochemical analysis and the presence of a mild damage by TEM, the possibility that DA exerts its effects through an indirect way, affecting the hippocampus, cannot be discarded. This brain region is responsible of seizure induction [53], and the alterations in the hippocampus are able to produce cardiac damage (arrhythmias) [54]. Vranyac-Tramoundanas *et al.* observed cardiac damage both in intrahippocampal and intraperitoneal administered rats, and these injuries were similar among two routes of administration [29]. Additionally, they only detect the toxin in circulation after i.p. administration, while they did not detect DA after intrahippocampal administration, discarding the possibility that DA was able to reach the heart [29]. Therefore, the damage observed in our experiments may be produced by the affectation of the DA main target, the hippocampus. Hence, is reasonable to think that DA is able to produce alterations in the cardiac tissue (directly or by an indirect way).

Cardiac alterations were observed in intoxication in human beings observed in 1987 in Canada. Afterwards this outbreak, in the year 1991 there were also reports of illness in some people after eating clams with DA presence [55]. Although no cardiac effects were observed in this second outbreak, this intoxication was mild compared to Prince Edward Island intoxication. Due to the confirmation of DA cardiotoxic effects, it would be desirable to continue the study of DA with new approaches or different doses in order to elucidate the real impact of the cardiac alterations.

## 4. Conclusions

Based on the present results, we can confirm the affinity of DA for heart tissue; in other words, this phycotoxin is able to cause injuries in this organ. Although optical microscopy did not show any observable cardiac damage, TEM showed the loss of myofibrillar arrangement and severe mitochondrial alterations. Moreover, the presence of DA in heart tissue, detected by immunohistochemistry, can be correlated with the described injuries.

## 5. Materials and Methods

### 5.1. Toxin Analysis

DA (5 mg) was purchased from Sigma-Aldrich (Munich, Germany). To verify the amount of DA provided in the commercial vial, toxin was analyzed using a Waters Alliance HPLC system 2695 (Waters Cromatografía, S.A., Cerdanyola del Vallès, Spain) separation module and a Waters 2487 UV/Vis detector (Waters Cromatografía, S.A., Cerdanyola del Vallès, Spain). Chromatographic separation was performed on a reversed-phase column C18 Kinetex (2.1 × 100 mm, 2.6 μm, 100 Å, Phenomenex, Madrid, Spain) protected by a krudkatcher ultra HPLC in-line filter (0.5 μm, Phenomenex). Isocratic elution 95:5 (A:B) was carried out with a mobile phase A consisting of water with 0.1% acetic acid and a mobile phase B consisting of acetonitrile. Flow rate was set at 0.4 mL/min and analysis time at 10 min. Column oven temperature was kept at 40 °C, sample manager was kept at 6 °C and injection volume was 10 μL .

Each dilution were injected in triplicate, and control samples including blank of reagents and DA standard solution (2 μg/mL) were also included within each sample set as quality control samples. External calibration was performed using the DA certified standard solution CRM-03-DA (CIFGA Laboratory, Lugo, Spain). Calibration curve range was performed from 0.06 μg/mL to 4.40 μg/mL through 8 concentration levels and in triplicate analysis. CRM-03-DA certified standard concentration is integrated by concentrations of DA and C5′-epi-domoic acid. Under these chromatographic conditions DA and C5′-epi-domoic acid are resolved, but only DA area is used to calibration, the low percentage of C5′-epi-domoic is assumed as quantization uncertainty.

The EmPower^®^ software (version 3, Waters, Milford, MA, USA, 2010) was used for the entire HPLC tune, instrument control, data acquisition, and data analysis.

### 5.2. Animals and Toxin Treatment

A total of 12 adult female Sprague-Dawley rats (weighing 180–215 g, mean weight 200 g) were employed in this study: 9 experimental and 3 control animals. Animals were individually housed, with a rodent diet and water provided *ad libitum*, and maintained at a controlled temperature (23 ± 2 °C) and humidity (60%–70%), under a 12 h light: dark cycle, for 1 week before the experiments and throughout the experimental period.

The dose of DA employed in the 9 treated rats was 2.5 mg/kg, as determined by previous experiments [44]. The experimental dose was chosen taken into account previous experiments [44], because it is able to cause injuries being under the LD_50_.

DA treated rats (*n* = 9) were inoculated i.p. with two doses of DA, spaced 30 days. The purpose of the second DA dose was to be able to detect DA presence in the organ, since this toxin is cleared as soon as 160 min after the administration [56]. Control animals (*n* = 3) were inoculated i.p. twice in the same way with physiological saline solution. The surviving experimental animals were sacrificed 1 h after the second inoculation of toxin with an i.p. overdose of sodium pentobarbital, and control specimens were sacrificed at the same time.

All the procedures using animals were conducted according to the principles approved by the Institutional Animal Care Committee of the Universidad de Santiago de Compostela and Xunta de Galicia (“Pharmacological studies with compounds of natural and synthetic origin”, procedure approved with code 011/14, dated 1 September 2014). All possible efforts were made to reduce animal suffering and minimize the number of animals used.

### 5.3. Behavioural Analysis

Rats were placed in an observation chamber 30 min before DA or saline administration. Immediately after the first DA administration, the specimens were returned to the observation chamber and their behaviour was observed for 6 h in a cycle of 5 min on and 10 min off, monitoring all behavioural responses during three different observation periods: 0 to 24 h, 24 h to 48 h and 48 to 72 h. Four treated rats developed severe clinical signs and died within a period of time between 5 and 90 min after the first DA administration. After second toxin administration, symptoms were also observed until sacrifice 1 h after DA administration.

### 5.4. Histopathology and Immunohistochemistry

After sacrifice, necropsy was done and heart was sampled and fixed by immersion in Bouin’s solution. All samples were embedded in paraffin according to standard laboratory procedures, and sections of 3 μm thickness were mounted onto silanized slides and dried overnight at 37 °C. Four non-consecutive sections of each animal were employed in each staining or immunohistochemical assay. The sections were stained with H & E for routine histological analyses .

For immunohistochemical assays, sections were deparaffinized with xylene and rehydrated through a graded alcohol series. To block peroxidase activity and prevent non-specific staining, 1 h of pretreatment with Dako Real Peroxidase-Blocking Solution (Dako, Barcelona, Spain) was performed. After removal of blocking reagents, sections were rinsed 3 times in PBS with 0.005% Tween 20 (Panreac Química SAU, Barcelona, Spain) and then incubated with a monoclonal primary antibody anti-DA, courtesy of Dr. Christopher T. Elliott, Institute of Agri-Food and Land Use (IAFLU), School of Biological Sciences, Queen’s University (Belfast, Northern Ireland, UK). The affinity of this antibody for the toxin was recently confirmed in our laboratory [57]. As negative controls, we have employed the control rats, which did not show any immunoreactivity. Additionally, other negative controls were carried out substituting the primary or the secondary antibody for PBS or an irrelevant polyclonal antibody. As positive controls we have employed muscular samples from rats of a previous experiment, where the toxin was intramuscularlly inoculated into the hind limb and the specimens were sacrificed 5 min after toxin administration.

The dilution employed was 1:5000 and the antibody was incubated overnight. Thereafter, sections were rinsed 3 times in PBS-Tween 20 and incubated with the secondary antibody solution (Dako REAL™ EnVisionTM Detection System, Dako, Barcelona, Spain) during 30 min. Samples were washed 3 times with PBS-Tween 20 and revealed with diaminobenzidine (DAB+ Chromogen, Dako, Barcelona, Spain). Finally, the slides were counterstained with haematoxylin, dehydrated, and permanently mounted in DPX (BDH Laboratory Supplies, Poole, UK).

### 5.5. Preparation of Samples for Transmission Electron Microscopy (TEM)

Heart samples (1 mm^3^) of control and treated specimens were fixed by immersion in 2.5% glutaraldehyde in 0.1 M cacodylate trihydrate buffer for 30 min at 4 °C in an orbital shaker at low speed. Fixative was then removed and the samples were rinsed three times with 0.1 M cacodylate trihydrate buffer. Post-fixation by immersion in 1% OsO_4_ in 0.1 M cacodylate trihydrate buffer was performed for 60 min. Finally, after a second rinse fixed tissues were dehydrated in graded ethanol solutions, including one bath with 70% ethanol and 0.5% uranyl acetate, rinsed in propylene oxide and embedded in Epon 812 (Momentive Specialty Chemicals Inc., Houston, TX, USA). A Leica Ultracut UCT ultramicrotome from Leica Microsystems GmbH (Wetzlar, Germany) was used to obtain ultrathin sections of tissue samples and they were counterstained with uranyl acetate and lead citrate. Ultrastructural analysis of 1 mm^2^ samples was performed with a JEOL JEM-1011 Transmission Electron Microscope (Jeol Ltd., Tokyo, Japan).

## Figures and Tables

**Table 1:** Summary of symptoms observed after first DA toxin administration (number of affected animals/*n* = 9).

Time	Hypoactivity	Head Shaking	Convulsions	Scratching	Hematoporphyrin Deposits	Absence of Water Intake (Adipsia)	Absence of Food Intake (Anorexia)	Death
0–24 h	9/9	9/9	9/9	9/9	0/9	7/9	8/9	4/9
24–48 h	0/5	0/5	0/5	1/5	2/5	3/5	4/5	0/5
48–72 h	0/5	0/5	0/5	0/5	0/5	1/5	2/5	0/5

## Abbreviations

The following abbreviations are used in this manuscript:

RIADT: Rede de Infraestruturas de Apoio á Investigación e ao Desenvolvemento Tecnolóxico
DA: Domoic acid
ASP: amnesic shellfish poisoning
KA: kainic acid
Glu: glutamate
GluRs: glutamate receptors
AMPA: alpha-amino-3-hydroxy-5-methyl-4-isoxazolepropionic acid
Ca^2+^: calcium
NMDA: *N*-methyl-d-aspartate
ROS: Reactive Oxide Species
CNS: Central Nervous System
i.p.: intraperitoneal
HPLC-UV: High-performance liquid chromatography-Ultraviolet/Visible
H & E: Haematoxylin and eosin
IHC: immunohistochemistry
TEM: transmission electron microscopy
M: mitochondria
IAFLU: Institute of Agri-Food and Land Use
FEDER: Fondo Europeo de Desarrollo Regional
CDTI: Centro para el Desarrollo Tecnológico e Industrial
AGL: Programa Nacional de Recursos y Tecnologías Agroalimentarias
ISIP: India & Spain Innovating Program
